# Serum Hsa_circ_0005962 Is A Prognostic Biomarker of Paclitaxel Resistance in Nonsmall Cell Lung Cancer Treatment

**DOI:** 10.1155/2023/6644168

**Published:** 2023-10-19

**Authors:** Chenliang Wang, Shaofeng Xia

**Affiliations:** ^1^Department of Pathology, The First People's Hospital of Jiujiang City, Jiujiang, Jiangxi, China; ^2^Department of Thoracic Surgery, The First People's Hospital of Jiujiang City, Jiujiang, Jiangxi, China

## Abstract

**Background:**

Tumor progression and the therapeutic resistance associated with cancer agents are thought to be modulated by circular RNAs (circRNAs); however, its mechanism associated with nonsmall cell lung cancer (NSCLC) is still undetermined. The following investigation aimed to evaluate the involvement of circRNAs with NSCLC.

**Methods:**

The serum specimens of 146 NSCLC individuals who received complete four cycles of PTX chemotherapy were collected. The serum concentration of hsa_circ_0005962 of these individuals was assessed with quantitative real-time polymerase chain reaction (qRT-PCR), followed by the evaluation of demographic and survival consequences for further assessments.

**Results:**

It was revealed that hsa_circ_0005962 is substantially increased in NSCLC chemoresistant patients and was positively correlated with the disease stage. Furthermore, the hsa_circ_0005962 value of the area under the curve was moderate, and increased hsa_circ_0005962 expression was linked with shorter overall survival (OS). Hsa_circ_0005962 stimulated paclitaxel resistance (PTX-R) in resistant NSCLC cells by regulating the axis of miR-126-5p/insulin-like growth factor 1 (IGF1).

**Conclusion:**

The results of this investigation highlight that hsa_circ_0005962 induces chemoresistance in NSCLC patients and, therefore, can act as a physiological target to treat NSCLC.

## 1. Introduction

Of all the different types of cancers, that of the lung is frequently occurring malignant tumors and its type; nonsmall cell lung cancer (NSCLC) is believed to account for 80% to 85% of lung cancers [1, 2]. Although the surgical strategies, imaging technologies, and radio- and chemo-therapeutics methods have developed significantly, the treatment efficiency associated with NSCLC is still unsatisfactory. This is because most NSCLC cases are diagnosed only at the terminal stage due to different reasons [3, 4]. Currently, for treating terminal-stage cancer, chemotherapy is mainly applied. However, drug resistance is the principal reason behind unsuccessful chemotherapy. Effective drug development and novel strategies that could prevent or determine drug-resistant cancers can effectively improve chemotherapy efficiency [5, 6].

Circular RNAs (circRNAs) are in the form of a loop, with the 3′ and 5′ ends covalently connected thereby forming a circle [7]. In molecular biology, endogenous RNA competition is based on miRNA's ability to control other RNA transcripts [8–10]. CircRNAs are important carcinogenesis and chemoresistant regulators [11, 12]. For instance, circ_0007331 stimulates colorectal cancer by regulating the high-mobility group A2 axis of miR-205-5p [13], circ-ABCB10 is linked with paclitaxel-resistance (PTX-R) in breast cancer via let-7a-5p/dual specificity phosphatase 7 (DUSP7) axis [14], and circ_ZFR also participates in PTX-resistance and NSCLC development by inducing karyopherin subunit alpha 4 (KPNA4) via miR-195-5p sponging [15]. Hsa_circ_0005962 is believed to have an oncogene activity and can enhance NSCLC progression [16]. However, hsa_circ_0005962 associations with chemoresistance of NSCLC individuals are still unexplored.

In this investigation, the circular RNA hsa_circ_0005962 locates at chr8:101936182-101937267, and its associated-gene symbol is YWHAZ. The serum concentration of hsa_circ_0005962 in NSCLC individuals and its correlation with clinical outcomes were determined. It was revealed that PTX-R NSCLC individuals had increased hsa_circ_0005962 expressions, which were associated with substandard overall survival (OS). Hsa_circ_0005962 induced NSCLC individuals PTX resistance by regulating miR-126-5p/IGF1 axis. Furthermore, hsa_circ_0005962 presented a reasonable area under the receiver operating characteristic (AUC-ROC) curve data, indicating its importance as a new prognostic bio-index of PTX-R NSCLC.

## 2. Materials and Method

### 2.1. Clinical Samples and Cell Culture

Serum specimens of 146 NSCLC and 142 healthy control individuals enrolled at First People's Hospital of Jiujiang were obtained. This investigation was authenticated by the ethical board of the First People's Hospital of Jiujiang, and all the subjects were initially informed about the research, and then, their signed consent was taken. Primary human bronchial epithelial (HBE) and NSCLC (A549 and H460) cells were acquired from Procell (Wuhan, China). The PTX-R corresponding NSCLC cells (H460-PTX and A549-PTX) was developed by augmenting the parental cells with accelerating PTX concentrations (SP8020; Solarbio, Beijing, China). All the cultures were propagated in RPMI1640 media (Invitrogen, Carlsbad, CA, USA) augmented with 10% fetal bovine serum (FBS; Invitrogen) and 1% penicillin–streptomycin (Invitrogen) at the standard temperature of 37°C and CO_2_ percentage of 5. Additionally, 5 nM of PTX (Solarbio) was introduced in the growth media to conserve H460-PTX and A549-PTX cell resistance.

### 2.2. Quantitative Real-Time Polymerase Chain Reaction (qRT-PCR)

Whole cellular RNA was procured via the RNeasy Mini kit (Qiagen, Valencia, CA, USA) and assessed by NanoDrop 2000c Spectrophotometer (Thermo Fisher Scientific, Waltham, MA, USA). Then, with the help of an M-MLV reverse transcriptase kit (Promega, Madison, WI, USA) or TaqMan MicroRNA reverse transcription kit (Applied Biosystems, Foster City, CA, USA), the cDNA was prepared, followed by qRT-PCR evaluation on the StepOnePlus Real-Time PCR System (Applied Biosystems) with SYBR Green PCR Master Mix (Invitrogen) and specified primers (RIBOBIO, Guangzhou, China). The primers used in this investigation were hsa_circ_0005962 forward: 5′AACTCCCCAGAGAAAGCCTGC3′ and reverse: 5′TGCTTGTGAAGCATTGGGGAT3′; IGF1 forward: 5′CGTCTCCCGTTCGCTAAATC 3′ and reverse: 5′AATAAAAGCCCCGGTCTCCA3′; miR-126-5p forward: 5′GCCGAGCATTATTACTTTT3′ and reverse: 5′CAGTGCAGGGTCCGAGGTAT3′;glyceraldehyde-phosphate dehydrogenase (GAPDH) forward: 5′AGAAGGCTGGGGCTCATTTG3′ and reverse: 5′AGGGGCCATCCACAGTCTTC3′; U6 forward: 5′GGAACGATACAGAGAAGATTAGC3′ and reverse: 5′TGGAACGCTTCACGAATTTGCG3′.

### 2.3. 3-(4,5-Dimethylthiazol-2-yl)-2,5-Diphenyltetrazolium Bromide (MTT) Analysis

After siRNA/plasmid was incorporated, 5,000 cells (H460-PTX and A549-PTX) were propagated in 96-well plates and then allowed 48 h of DTX, DDP, and PTX exposure. Thereafter, MTT reagent (2 mg/mL) (Sigma-Aldrich) was introduced to react with cells for 4 h. Living cells formed formazan, and these were then resolved in dimethylsulfoxide (100 *μ*l). Lastly, the absorbance of cells (470 nm) was determined via a microplate reader. IC_50_ of DTX, DDP, and PTX was assessed on GraphPad Prism 7 software (San Diego, USA).

### 2.4. Statistical Evaluation

All the protocols were performed thrice, and their data were assessed with GraphPad Prism 7 and depicted by values of the mean of ± standard deviation. Student's *t*-test and one-way analysis of variance (ANOVA) were carried out for differential analysis. *P* value <00.5 was termed significant.

## 3. Results

### 3.1. NSCLC Patients Exhibit Upregulation of Serum Hsa_circ_0005962

**Figure 1:**
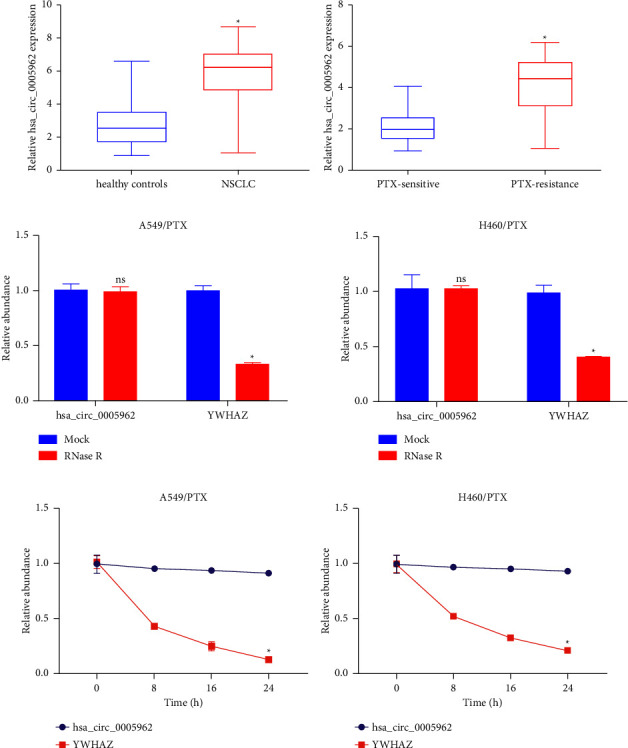
NSCLC patients exhibit serum hsa_circ_0005962 upregulation. (a) Serum hsa_circ_0005962 levels were higher in NSCLC patients. (b) PTX-resistant patients exhibited higher levels of hsa_circ_0005962 expression compared with PTX-sensitive patients in the before and after treatments (*n* = 80). (c) The expression of hsa_circ_0005962 and YWHAZ mRNA after treatment with RNase R in A549/PTX and H460/PTX cells. (d) The expression of hsa_circ_0005962 and YWHAZ mRNA after treatment with actinomycin D at the indicated time points in A549/PTX and H460/PTX cells. ^*∗*^*p* <  0.05.

To assess the efficiency of hsa_circ_0005962 as a physiological chemoresistance index, its serum concentration was assessed. NSCLC individuals showed notably increased serum hsa_circ_0005962 expression than control individuals (Figure 1(a)). Moreover, its expression was greater in PTX-R (*n* = 64) than in PTX-sensitive patients (*n* = 82) (Figure 1(b)). The data indicated the efficacy of this circRNA as an index for NSCLC chemotherapy. In addition, resistance to digestion with RNase R exonuclease specifically degraded linear RNAs but not circRNAs (Figure 1(c)). The results of actinomycin D assays revealed that the half-life of the hsa_circ_0005962 transcript exceeded 24 h, longer than the half-life of YWHAZ mRNA, indicating that hsa_circ_0005962 is more stable than the linear YWHAZ transcript (Figure 1(d)).

### 3.2. Hsa_circ_0005962 Expressions Are Related to NSCLC Patients' Clinical Manifestations

**Figure 2:**
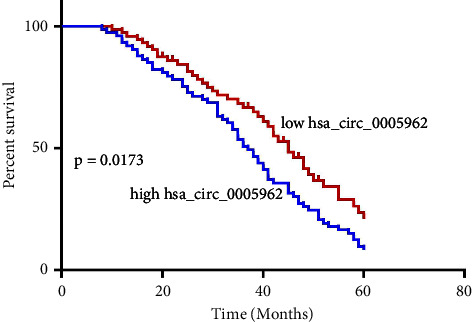
Hsa_circ_0005962 levels are related to clinical features in NSCLC patients. Patients exhibiting higher hsa_circ_0005962 expression levels exhibited prolonged overall survival compared with patients with lower levels.

Next, the NSCLC individuals were stratified into groups with high and low hsa_circ_0005962 concentrations in serum, according to their average expression in this cohort. Furthermore, the clinical manifestations were also compared between the two cohorts. Chi-squared analyses indicated the association of hsa_circ_0005962 expressions with tumor size, TNM stages, distant metastasis or recurrence, and lymph node metastasis (Table 1); however, it was negatively related to age, gender, or tumor type. Kaplan–Meier assessment suggested that individuals who exhibit enhanced hsa_circ_0005962 expression have shorter OS than those with low expression levels (Figure 2).

### 3.3. Hsa_circ_0005962 Is Associated with Substandard Prognosis of Chemoresistant NSCLC Individuals

**Figure 3:**
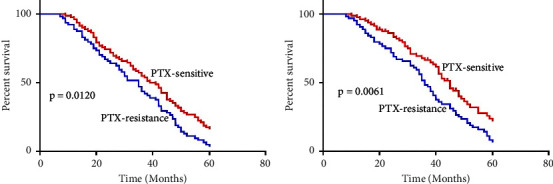
Hsa_circ_0005962 is associated with poor chemoresistant NSCLC patient prognosis. Chemoresistant NSCLC patients exhibited significantly decreased progression-free survival (a) and overall survival (b) relative to chemosensitive patients.

Kaplan–Meier and log-rank tests revealed that chemoresistant NSCLC individuals have substantially decreased OS and progression-free survival (PFS) than chemosensitive individuals (Figure 3). Cox proportional hazards regression analyses indicated the association of hsa_circ_0005962 levels with tumor size, TNM stages, distant metastasis or recurrence, lymph node metastasis, and chemoresistance of patient PFS (Table 2) and OS (Table 3), emphasizing the importance of this circRNA as an independent predictor of survivability of chemoresistant NSCLC patients.

### 3.4. Serum hsa_circ_0005962 Concentrations Are a Diagnostic Index for NSCLC Chemoresistance

**Figure 4:**
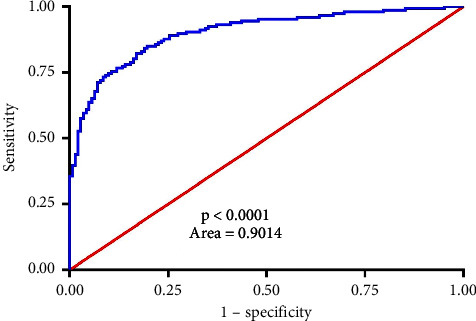
Serum hsa_circ_0005962 levels offer diagnostic utility for the detection of NSCLC chemoresistance. Receiver-operating characteristic curves were used to differentiate between chemoresistant NSCLC patients before and after therapy.

To determine the diagnostic efficiency of concerned circRNA in serum, the AUC-ROC was assessed and observed to be 0.9014 (95% CI, 0.8661–0.9367, Figure 4, *p* <  0.0001), compatible with its application as a physiological marker for differentiating NSCLC suffering individuals from healthy subjects.

### 3.5. Hsa_circ_0005962 Contributes to the PTX Resistance of NSCLC by miR-126-5p/IGF1 Axis

**Figure 5:**
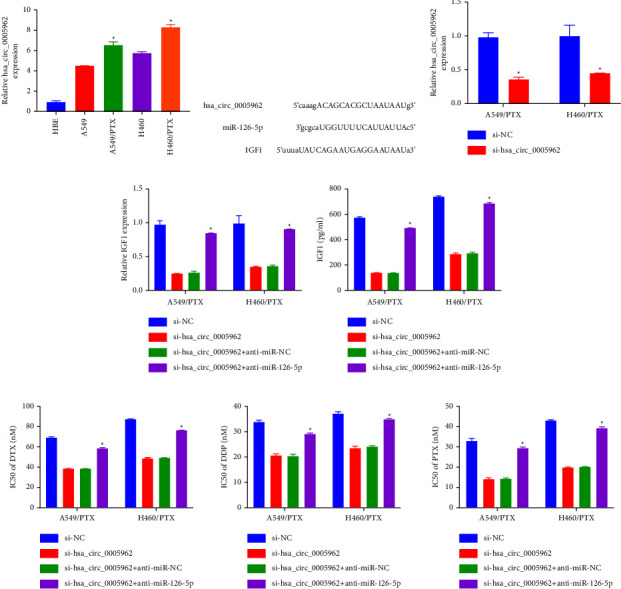
Hsa_circ_0005962 contributes to the PTX resistance of NSCLC by the miR-126-5p/IGF1 axis. (a) The has_circ_0005962 level in A549 and H460 cells was higher than that in HBE cells and lower than that in A549/PTX and H460/PTX cells. (b) RT-qPCR assay for has_circ_0005962 expression in A549/PTX and H460/PTX cells transfected with si-NC or si-has_circ_0005962. (c) Potential binding sites within hsa_circ_0005962, miR-12,6-5p, and IGF1 were researched by using STARBASE 3.0. (d, e) IGF1 expression was determined by qRT-PCR and Elisa. (f) IC50 of DTX, DDP, and PTX was estimated by MTT assay. ^*∗*^*p* < 0.05.

Hsa_circ_0005962 expressions in A549 and H460 cells were greater than those in HBE cells and less than in H460-PTX and A549-PTX cells (Figure 5(a)). We successfully constructed knockdown hsa_circ_0005962 H460-PTX and A549-PTX cells (Figure 5(b)). To determine mechanical hsa_circ_0005962 pathways, potential attachment sites within miR-126-5p, hsa_circ_0005962, and IGF1 were searched via STARBASE 3.0 (Figure 5(c)). The established resistant cell lines were transfected with si-hsa_circ_0005962, si-NC, si-hsa_circ_0005962 + anti-miR-NC, and si-hsa_circ_0005962 + anti-miR-126-5p for further determining the relationship among the aforementioned binding sites. Hsa_circ_0005962 silencing substantially decreases the IGF1 expression (Figures 5(d) and 5(e)) and IC_50_ of DTX, DDP, and PTX (Figure 5(f)) in resistant cells, whereas anti-miR-126-5p potentially reversed this effect.

## 4. Discussion

Most of the chemotherapeutic agents fail to successfully treat cancer because of chemoresistance in humans, including resistance against NSCLC. Advancements in highly efficient sequencing technology have allowed the discovery of diverse circRNAs that regulate chemoresistance development. NSCLC individuals' serum indicated substantially increased hsa_circ_0005962 levels relative to the control cohort. Furthermore, hsa_circ_0005962 upregulation in PTX-R NSCLC individuals relative to chemosensitive individuals indicated its importance as an independent predictor of clinical outcomes. In addition, hsa_circ_0005962 stimulated PTX-R by controlling miR-126-5p/IGF1 axis.

The importance of circRNAs activity in NSCLC development and drug resistance has slowly gained a lot of attention from scientists. For example, the circ-CPA4/let-7 miRNA/PD-L1 axis modulates the propagation, stemness, immune evasion, and drug resistance of NSCLC cells [17]. Suppressing circ_0014130 inhibits resistance against drugs and the malignant function of docetaxel-resistant NSCLC cells by controlling the axis of miR-545-3p-yes-associated protein 1 (YAP1) [18]. The hsa_circ_0002874 overexpression induces PTX resistance in NSCLC by controlling miR-1273f/murine double minute 2 (MDM2)/p53 pathway [19]. These studies indicate the important function of circRNAs in drug resistance in NSCLC. Additionally, circRNAs are also excellent physiological markers such as circ_0001649 acts as a marker by inhibiting NSCLC progression by miR-331-3p and miR-338-5p sponging [20], hsa_circ_0033155 is also a novel NSCLC index [21], and hsa_circ_0102533 is a blood-based marker for diagnosing NSCLC and regulating in vitro apoptosis [22].

In this investigation, the hsa_circ_0005962 expressions were elevated in both PTX-R and PTX-sensitive NSCLC individuals, indicating its importance as a valuable predictor of chemotherapeutic responses. Hsa_circ_0005962 concentrations were associated with tumor size, TNM stages, distant metastasis or recurrence, and lymph node metastasis in NSCLC individuals and were not related to age, gender, and tumor type. Kaplan–Meier method suggested that increased hsa_circ_0005962 expressions were linked with short OS compared with low levels. Furthermore, chemoresistant subjects had shorter OS and PFS than chemosensitive subjects. Univariate and multivariate analyses suggested that tumor size, TNM stages, distant metastasis or recurrence, lymph node metastasis, and chemoresistance were correlated with PFS and OS, indicating that hsa_circ_0005962 is an effective independent outcome predictor for NSCLC individuals. Additionally, the AUC value (0.9014) indicated that serum expression of hsa_circ_0005962 can be reliably utilized for differentiating NSCLC individuals from healthy subjects.

## 5. Conclusion

This investigation summarizes that the upregulation of hsa_circ_0005962 is prominently evident in the NSCLC individuals' serum samples, where this upregulation was substantially pronounced in the serum of chemosensitive rather than chemoresistant subjects. Furthermore, hsa_circ_0005962 promoted PTX resistance in PTX-R NSCLC cells by modulating miR-126-5p/IGF1 axis. Therefore, hsa_circ_0005962 is an effective target that opens a new path for further studies to evaluate the underlying processes involved in NSCLC chemoresistance.

## Figures and Tables

**Table 1:** Correlations between hsa_circ_0005962 levels and NSCLC patient clinicopathological features.

Variables	Low hsa_circ_0005962 expression	High hsa_circ_0005962 expression	*P* value
Age (years)			0.546
≤60	37	39	
>60	36	34	
Gender			0.236
Male	56	54	
Female	17	19	
Tumor size (cm)			<0.05
≤3	52	13	
>3	21	60	
Tumor type			0.195
Squamous cell carcinoma	43	45	
Adenocarcinoma	30	28	
TNM stages			<0.05
I-II	49	22	
III-IV	24	51	
Distant metastasis or recurrence			<0.05
Negative	51	27	
Positive	22	46	
Lymph nodes metastasis			<0.05
Negative	47	25	
Positive	26	48	

**Table 2:** Univariate and multivariate analyses of NSCLC patient progression-free survival.

Variables	Univariate analysis		*p* value	Multivariate analysis		*p* value
	HR	95% CI		HR	95% CI	
Age (years)	0.523	0.125–1.254	0.657	—	—	—
Gender	0.615	0.264–1.542	0.419	—	—	—
Tumor size (cm)	2.512	1.024–3.025	0.036	1.956	0.952–2.856	0.038
Tumor type	0.746	0.295–1.954	0.471	—	—	—
TNM stages	2.195	1.225–3.251	0.022	2.012	1.025–2.954	0.031
Distant metastasis or recurrence	1.965	0.619–2.652	0.028	1.572	0.754–2.421	0.035
Lymph nodes metastasis	2.415	0.954–3.652	0.021	2.149	0.719–3.095	0.029
hsa_circ_0005962 expression	2.951	1.115–3.425	0.008	2.546	0.849–3.152	0.011
Chemoresistance	3.024	1.845–3.952	0.012	2.952	1.528–3.586	0.019

**Table 3:** Univariate and multivariate analyses of NSCLC patients' overall survival.

Variables	Univariate analysis		*p* value	Multivariate analysis		*p* value
	HR	95% CI		HR	95% CI	
Age (years)	0.692	0.215–1.365	0.719	—	—	—
Gender	0.635	0.341–1.846	0.585	—	—	—
Tumor size (cm)	2.754	1.254–3.485	0.039	2.415	0.855–2.952	0.034
Tumor type	0.815	0.154–1.955	0.519	—	—	—
TNM stages	2.854	1.842–3.854	0.028	1.854	0.955–2.546	0.028
Distant metastasis or recurrence	2.015	0.842–2.954	0.034	1.485	0.628–2.854	0.038
Lymph nodes metastasis	2.365	0.854–3.652	0.028	1.952	0.846–2.954	0.034
Hsa_circ_0005962 expression	3.142	1.354–4.021	0.007	2.258	1.248–3.645	0.015
Chemoresistance	3.252	1.485–4.125	0.019	2.854	1.341–3.842	0.022

## Data Availability

The data used to support the findings of this study are available from the corresponding author upon request.
